# Contextually Appropriate Tools and Solutions to Facilitate Healthy Eating Identified by People with Type 2 Diabetes

**DOI:** 10.3390/nu13072301

**Published:** 2021-07-03

**Authors:** M. Carolina Archundia Herrera, Denise L. Campbell-Scherer, Rhonda C. Bell, Catherine B. Chan

**Affiliations:** 1 4-102 Li Ka Shing Centre for Health Innovation Research, Department of Agriculture, Food and Nutritional Science, Alberta Diabetes Institute, University of Alberta, Edmonton, AB T6G 2E1, Canada; archundi@ualberta.ca (M.C.A.H.); bellr@ualberta.ca (R.C.B.); 2 Office of Lifelong Learning & the Physician Learning Program, Department of Family Medicine, Alberta Diabetes Institute, University of Alberta, 2-590 ECHA, Edmonton, AB T6G 1C9, Canada; dlcampbe@ualberta.ca; 3 6-002 Li Ka Shing Centre for Health Innovation Research, Department of Physiology, Alberta Diabetes Institute, University of Alberta, Edmonton, AB T6G 2E1, Canada

**Keywords:** type 2 diabetes, lifestyle intervention program, healthy eating, qualitative

## Abstract

Type 2 diabetes (T2D) is a complex, multifaceted disease and its treatment involves lifestyle intervention (LI) programs that participants may find difficult to adopt and maintain. The objective of this study is to understand the lived experiences of participants with T2D regarding healthy eating behavior change, in order to identify and incorporate relevant information, skills, and educational approaches into LI programs. An explorative qualitative study was undertaken. Purposeful sampling was used to recruit 15 participants. One-on-one, semi-structured, open-ended, and in-depth interviews were conducted. An essentialist paradigm was adopted to accurately report the experiences, meaning, and reality of participants. An inductive approach was used to analyze the data. Participants reported that being diagnosed and living with T2D could be overwhelming, and their ability to manage was influenced by health care providers (HCP), family, and individual context. Many experienced a loop of “good–bad” eating behaviors. Participants expressed desires for future diabetes management that would include program content (nutrition, physical activity, mental health, foot care, and consequences of T2D), program features (understand context, explicit information, individualized, hands-on learning, applicable, realistic, incremental, and practical), program components (access to multidisciplinary team, set goals, track progress and be held accountable, one-on-one sessions, group support, maintenance/follow-up), and policy change. In conclusion, the results of this study indicate that T2D management requires more extensive, comprehensive, and ongoing support, guided by the individual participant.

## 1. Introduction

Type 2 diabetes (T2D) is a complex disease driven by multiple pathophysiological processes [1]. Thus, a multidisciplinary approach that integrates the four main pillars of T2D management including self-management education (SME) and support (SMS), nutrition therapy (NT), physical activity (PA) and pharmacological therapy is recommended to achieve optimal glycemic management, thereby minimizing disease complications [2]. However, incorporating these recommendations into daily life represents a heavy, sometimes overwhelming burden on patients, who need to implement multiple treatment strategies including adherence to medications, as well as changes in eating habits and physical activity.

Lifestyle interventions (LI) that include change in diet and PA as well as education help guide participants to implement changes, resulting in significant improvement in cardiovascular disease risk factors [3], clinical outcomes [4,5], improved beta cell function [6], self-management practices, and quality of life (QOL) [7]. Through a process of SME and SMS people with T2D can acquire the motivation, knowledge, and skills necessary to manage blood glucose [8].

Short-term effectiveness of LI programs to manage T2D has been documented in high [9,10], middle, and low income countries [11], and ethnic minorities [12] but their long-term effects are often attenuated [9,10,11,12], pointing to a gap in knowledge between what people learn in the programs and their actions over the long run [13]. This is especially true for NT. As reflected in the Diabetes Canada NT Guidelines [14], transitioning to a healthier eating pattern may necessitate several behavior modifications and acquisition of food skills to adequately understand the recommendations and to select, plan, prepare, and store meals and snacks. Thus, this behavior is especially hard to achieve and sustain over decades [15]. There is a lack of research aimed at identifying which of the NT guidelines are particularly challenging to adopt and which facilitators could be potential intervention components. In addition, the global increase in access to ultra-processed foods [16] supports busy lifestyles, but diminishes food skills value and importance, consequently, home-prepared meals using whole ingredients and cooking skills are declining [17]. Several studies have aimed to identify barriers and facilitators to sustained self-management of T2D [18,19]. However there is an unequivocal imbalance between facilitators and barriers in the interview guides used [20], for example, the term “facilitator” is used interchangeably with less specific words (e.g., “help you” or “easier”) and is usually mentioned only once or twice, whereas the term “barrier” is mentioned literally and several times in the interviews [18,21,22,23]. This prejudices the study to identify barriers more than facilitators. Thus, little is known about how to facilitate healthy eating, especially from the participants’ perspectives. The aim of this study is to work with T2D participants to understand their perceptions and lived experiences regarding healthy eating behavior change, enabling the identification and incorporation of relevant intervention components into T2D LI programs.

## 2. Materials and Methods

### 2.1. Design

Given the general lack of understanding on facilitators of behavior modification for healthy eating in LI, an explorative qualitative study was undertaken to understand the lived experience of people with T2D, what healthy eating habits meant to them, and how they solved some of the barriers they encountered when implementing healthy eating behaviors. To achieve this aim, purposeful sampling was used. This method allowed for selection of information-rich cases (thus, those individuals who could provide valuable information towards our aim), enabling and yielding insights and in-depth understanding on how to facilitate healthy behavior change [24]. Additionally, participants were recruited from different age groups, gender identifications, socioeconomic backgrounds, and ethnocultural affiliations to maximize variations of participant context as much as possible. The COREQ checklist was used as a guide when reporting the study methods (Supplementary File S1) [25].

### 2.2. Participants

The study took place in the Human Nutrition Research Unit at the University of Alberta, Edmonton, AB, Canada (prior to April 2020) and online using secure, encrypted videoconferencing software (necessitated by COVID-19 restrictions from April to September 2020). Adults (18+ years) diagnosed with T2D (self-identified) who had participated in LI programs, had past consultations with a registered dietitian (RD), and/or any other health care provider (HCP) regarding their T2D management, and who were able to speak/write English were included in this study. Individuals were not eligible to participate if they met any of the following criteria: (i) not able to read or write English because they needed to read the information provided and grant written consent; furthermore, participants were provided with a summary of the key messages from the Diabetes Canada NT Guidelines, thus, reading English was essential, and the interview was also conducted in English; (ii) individuals without T2D; (iii) pregnant or with medical comorbidities requiring a highly specialized diet (e.g., gastrointestinal or renal diseases, food allergies).

Participants were recruited from the 5As Team Intervention Study (5AsT) cohort [26] and the Alberta Diabetes Institute (ADI) Research Contact Registry. In both cases, the original databases were searched to identify participants with a T2D diagnosis. Access to their sex, age, and cultural background were also provided. Once identified, those from the ADI Research Contact Registry were sent information about the study by an administrator and asked if they were interested in participating. Then, the list of possible participants was provided to MCAH who approached them via telephone, as were individuals from the 5AsT cohort. In this first contact, MCAH explained the purpose of the study in detail, as well as the procedure and time requirements, and then answered questions and invited participants to participate (Supplementary File S2). Those interested were provided with an information letter containing all the details of the study and researchers’ contact information. A total of 82 potential participants were obtained from the two databases, 39 were invited to participate, 10 declined due to lack of time, and 14 agreed but later declined to participate due to COVID-19 concerns or difficulties connecting to the video-conference interview; thus, 15 consented to participate. The study was approved by the Research Ethics Board of the University of Alberta (Study ID: Pro00092713). All participants provided written informed consent prior to their interview. Parking costs were reimbursed for those attending in-person interviews.

### 2.3. Procedure

One-on-one, semi-structured, open-ended, in-depth interviews were conducted. Patton’s guide [24] was used to develop the interview guide. A total of 10 questions were asked in order to understand participants’ experiences in relation to healthy eating habits. The interview aimed to understand their overall knowledge and the importance healthy eating played in their lives, for example, “How important is healthy eating to you when you think about your diabetes?”; their past experiences in relation to healthy eating, “Describe to me any strategies you have used to help you eat healthy in the past?” and how to facilitate people’s adherence to the Diabetes Canada NT Guidelines [2] particularly focusing on behaviors that were potential targets for LI intervention, “What type of activities could we offer to help people make these changes?” Participants were also presented with a summary of the following key messages from the Diabetes Canada NT Guidelines to better understand the behavior and identify possible intervention options.

- Select whole and less refined foods instead of processed foods, such as sugar-sweetened beverages, fast foods and refined grains.
- Reduce caloric intake to achieve and maintain a healthier body weight.
- Pay attention to both carbohydrate quality and quantity.
- Select unsaturated oils and nuts as the preferred dietary fats.
- Choose lean animal proteins. Select more vegetable protein.

Cues and probes were used to clarify and obtain additional data. Journal notes were taken during and after each interview to record the researcher’s reflections, feelings, and interpretations [24].

Interviews were conducted by a female author (MCAH-RD, MSc, PhD candidate) with previous research experience with this population and in conducting focus groups. The reason for conducting the study and professional background of the interviewer were presented to the interviewees before the start of the session. Interviews were audio recorded (with participant’s consent). Three pilot interviews were carried out to ensure coherence and flow of questions, after which adjustments were made to the interview guide (Supplementary File S3). Participants were asked to complete a basic demographic questionnaire (Supplementary File S4).

### 2.4. Data Analysis

Data were collected and analyzed concurrently. All interviews were transcribed verbatim by an outsourced transcription service (Consentia Inc., Edmonton, AB, Canada) and subsequently checked against the audio recording for accuracy by MCAH. Each transcription page contained a header with the participant code (name removed and replaced with a number), the number of the interview, the location and date of the interview, and the page number. The data collected during the interviews was managed, analyzed, and coded using NVivo 12.6 (QSR International Pty, Ltd., Doncaster, Australia).

Thematic analysis was used for identifying, analyzing, and reporting patterns within the data [27]. An essentialist paradigm was adopted in order to report experiences, meanings and the reality of participants [27,28]. An inductive approach was adopted, thus, no predefined outcomes were established, and instead, the coded categories were derived directly from the data. The process of data analysis was guided by Vaismoradi (2013) and Braun & Clarke (2006) [27,28]. To ensure rigor, two independent coders (MCAH, CBC) coded 4 interviews using open coding, enabling the creation of initial codes from which 2 independent coding manuals were created. CBC (PhD, Professor of Human Nutrition) was trained to conduct the analysis by MCAH. MCAH and CBC compared and discussed the similarities to create a final coding manual. The rest of the interviews were coded by MCAH, providing full and equal attention to each transcript and refining codes as needed. MCAH defined categories and subsequently checked for convergence and divergence with CBC to create the final categories.

## 3. Results

The results of this study are organized as follows: In the first section, we describe participants’ demographic characteristics, backgrounds, and general health (Table 1), followed by a description of the programs and strategies they reported in their journey to manage T2D (Table 2). Subsequently, the 5 main themes developed from the analysis are described (Table 3, Table 4 and Table 5).

### 3.1. General Characteristics, Background, and Health

A total of 15 participants aged 33–79 years were interviewed (mean age 61 years). Participants had on average 12.6 years of T2D diagnosis varying from 5–25 years. Participants also varied in gender, ethnicity, education, and socioeconomic status (Table 1).

Most participants were from the greater Edmonton, Alberta area, but many had moved around previously for education or work. A wide variety of professions including taxi drivers, librarians, full time parents, laborers, HCPs, and bureaucrats was represented. The majority of the participants reported having concomitant health issues; “I broke my back in ’08 so I have challenges with that”—PT153, “I’ve gone through, I’ve gone through prostate cancer”—PT132, “I got arthritis in my knees”—PT98. Several participants shared having mental health issues: “I was d-diagnosed with severe depression”—PT178, “… my GP called it anxiety depression. Later on it was diagnosed as PTSD”—PT97. Some identified having obesity from an early age as a health issue, “… as a child… I was, uh, I was always overweight”—PT97; others linked obesity to childbirth, “I gained a lot with the first child”—PT38, “I had gained a tremendous amount of weight with that pregnancy”—PT623; others linked it to pharmaceutical therapy, especially with the use of insulin: “I’ve been gaining more weight—just diabetes and medication I’m taking. Insulin and all the pills. I’m just, like, exploding”—PT153.

As expected, the majority of participants had received a variety of T2D management advice from HCPs (general practitioner and specialist MDs, registered nurses, RDs, pharmacists, etc.). They had also attended various educational programs offered at hospitals, primary care settings, university research centers, and enrolled in commercially available programs (Jenny Craig, Inc., Weight Watchers International, Inc.). These programs focused on different pillars of T2D management (Table 2).

#### 3.1.1. Theme 1: Dealing with Being Diagnosed and Living with T2D

The analysis revealed a pattern of participants reporting being scared, confused, and terrified when diagnosed with T2D; “I was really overwhelmed with this. It was really, really difficult … you don’t know what’s happening with your body and it can be really confusing”—PT153. Many were overwhelmed by the process of living with T2D and felt anxious, trapped or ashamed; “I was just trapped—I was panicked, I was like no! I don’t even know which food is good for me right now’”—PT153, thus making eating an ongoing struggle. Those patterns identified dealing with T2D as a major theme of this analysis (Table 3).

Feeling accountable and engaged positively influenced and impacted how participants dealt with being diagnosed and living with T2D. Some participants were motivated and felt accountable to deal with T2D so they would not be a burden to others and to avoid developing further health issues; “A guy was there with a wheelchair, and […] we started chatting and then he said ‘yeah, I lost both my legs to diabetes.’ And it was like… it just… it—it impacted me. It, uh… I sat there afterwards looking at him sitting in the chair and he had no legs below his knees. And he wasn’t much younger—uh, older than me—uh, he was younger than me. And I thought about it and he’s now trapped wheelchair for the rest of life because he didn’t monitor and he said to me, he said ‘I didn’t take it seriously […] that’s a big motivator”—PT178. Achieving a good quality of life was a reason for dealing with T2D, as were life goals and awareness of future consequences. Participants noted that making an effort to be mindful, practicing self-management, and taking ownership of one’s health were ways to deal with T2D (Table 3).

#### 3.1.2. Theme 2: Impact of External Forces on T2D Management

Participant’s individual context influenced the way they were able to manage their disease, making this a theme that reflected the impact external forces and their life situation had on their T2D management. HCPs played a major role in providing patients with adequate guidance and tools to enable successful management. Participants reported being provided access to programs, updated information and ideas, and felt encouraged to adhere to the guidance provided when their HCPs were encouraging, enthusiastic, practically-oriented, positive, and supportive; “What I liked about her [RD] is she never told—like, ‘cause there’s things I don’t wanna eat. I hate avocado. I don’t care if it’s good for me. That stuff’s disgusting. You know, and so… um, she would like ‘okay, so try it’, so I would try it and then she says ‘okay, so you still don’t like that, why don’t we go for tomatoes instead and just add extra olive oil into your eggs?’ You know, she would make real suggestions that worked for me”—PT163 (Table 3). In contrast, for those who had difficulties accessing a HCP, or who encountered a HCP who did not provide enough time, attention or clear information, as well as those who did not support different treatment strategies and in general did not listen to them, the negative experiences had a direct detrimental impact on the participant’s T2D self-management; “I went to a doctor first. And he was actually, he–he told me to just try and lose weight. I don’t think it was a good thing to see the doctor […] and he didn’t really give me any indication about the diabetes, what I was supposed to do, like, he just told me to go back home and to lose weight and I was like ‘okay.’ So, I went back home and didn’t try anything”—PT153 (Table 3).

Family also had a big influence on how participants managed their disease. Having a family member who had experienced complications encouraged them to avoid going down that road; “I remember- remember when I was younger, my father had kicked a table in the dark with his foot and—his toe. And he didn’t go to the doctors right away ‘cause he was stubborn and cranky and he almost lost his foot because of the diabetes […]—I remembered that and I didn’t want to end up that way”—PT178. Household members who provided encouragement, guidance, and support motivated and enabled a positive attitude towards T2D management; “… my granddaughter, because she’s overweight as well and she’s 12, we’re doing 30 min on the treadmill at night together so, you know, she’s in another house, I’m in this house so we just kind of FaceTime and we just do our 30 min together. So even though I’m tired, she said ‘let’s go grandma’, you know, so we’re doing it together”—PT149. On the contrary, loss of a family member and family strains had a direct negative effect and led to discouragement and a surge in apathetic feelings. Participants also acknowledged that they compromised their own desire to eating healthy in order to harmonize meals with their family members. Becoming parents also hindered T2D management as a result of lack of time and having children who were picky eaters, which required preparing different meals; “Adopting the changes—I mean it gets harder as your kids are little and you keep having more children, I mean, life gets crazy”—PT623 (Table 3).

Several major factors that affected participants’ T2D management were directly influenced by their everyday context including time, personal resources, and competing priorities. Time and type of work impacted the quality and type of food they consumed; “I work in the oil field. And… I guess… something that can probably contribute to… the—the fluctuations I see is I don’t, um… I’m away from home a lot so I have to eat, um… restaurant food. And… it’s not that I would probably get a… a decent meal at 6 o’clock, it might be 8 or 9 o’clock”—PT300, several expressed views of healthy food being expensive, not affordable for everyone, and laborious to prepare whereas “junk food” was seen as easy, cheap and available; “Eating well does cost more money. It is very difficult to eat well. Uh, it’s easier to buy macaroni and cheese, you know?”—PT97. Changes in living arrangements, such as moving from their parents’ homes to living by themselves, or having children move out, or having one’s partner pass away also affected the way participants managed their T2D. On the one hand, having someone at home who took care of the planning, shopping, and cooking could lead to a lack of these skills when that person moved out, resulting in increased consumption of ready-to-eat foods. On the other hand, cooking for one after being used to cooking for more people was described as too much work and not worth the effort. The food environment, exemplified as widespread availability of fast food and processed food, coupons, TV advertisements, and promotions/discounts explained in part the lack of adherence to T2D management; “… in [grocery store] last night they had 10 different baked items. We could have chosen any of those for half price”—PT87 (Table 3).

The current, unprecedented COVID-19 situation had a negative impact on T2D management. Having gyms, recreation centers, and pools closed down triggered reductions in PA among these participants despite classes being offered online. Participants expressed strain in going out for walks, “… like some people don’t wanna go to the gym. They don’t wanna go for a walk and now with Covid, everybody’s scared to go outside”—PT178. Isolation from family, friends, and work was hard and stressful to adapt to (Table 3).

#### 3.1.3. Theme 3: Understanding Healthy Eating Behaviors for T2D Management

One aim of the interview was to obtain an understanding of what people perceived by “healthy eating habits”. Participants expressed their understanding in a variety of terms. They talked about types of foods (fruit and vegetables, fish, poultry, red meat, white flour, whole wheat flour, pasta, and sugar), nutrients (proteins, carbohydrates, fats, and fiber), state (natural, organic, processed food, artificial ingredients, and junk food), perceived value (nutritious foods, empty calories, good food, and bad food), and in terms of how to eat healthy (follow Canada’s Food Guide, use the plate method, limit calorie intake or portion size, less processed food, and incorporating meal planning) (Table 3).

Some participants described eating healthy with antagonism, referring to it as something they had to do, but did not enjoy; “I didn’t really like... fruits—or, uh, vegetables, really that much, before and now I just… consider it a… a requirement that I don’t exactly overly enjoy but it’s—it’s something that… I need to do, basically, right?”—PT300. Healthy eating was mentioned in terms of knowing what they should not be eating but uncertainty of what they should be eating; “I have a sense of some things I shouldn’t be eating but what should I be eating?”—PT555, also, how much to eat, and when (time of day) (Table 3). When looking at the specific knowledge and understanding of the key messages from the Diabetes Canada NT Guidelines presented to participants, a notable gap in knowledge emerged, in that people understood the guidelines, but were uncertain and hesitant as to how to follow them; “It talks about ‘sugar-sweetened beverages’, I understand that very clearly. Don’t—don’t eat those, you know, don’t—but is—is diet—is, uh, diet Pepsi okay or diet… you know, the diet drinks—are they good? Or should- should it say specifically ‘don’t drink sugary drinks, don’t drink… don’t drink, uh, pops and such. Drink waters, juices or these things much…’ you know, much clearer recommendations. That’s really where I struggle. I just don’t understand what I should be and shouldn’t be…”—PT555. This was accentuated in the key messages that used technical language such as unsaturated oils, glycemic index, and dietary patterns; “unsaturated oils.’ Um, so by ‘unsaturated oils’, are we talking, like, olive oil and that kind of thing?”—PT300 (Table 3). Furthermore, the thematic analysis revealed a pattern of participants not matching their actions or behavior with their knowledge; “I’ve got lists of things you’re supposed to eat and all the good things you’re supposed to have. And you go there. Do I shop with that list? I think about it. We try and do it, but not necessarily”—PT87. Many also reported going through cycles of “good behavior” and “bad behavior”. Those patterns make understanding healthy eating behaviors for T2D management a major theme of the study (Table 3).

#### 3.1.4. Theme 4: Understanding Needs and Identifying Intervention Components

**Figure 1:**
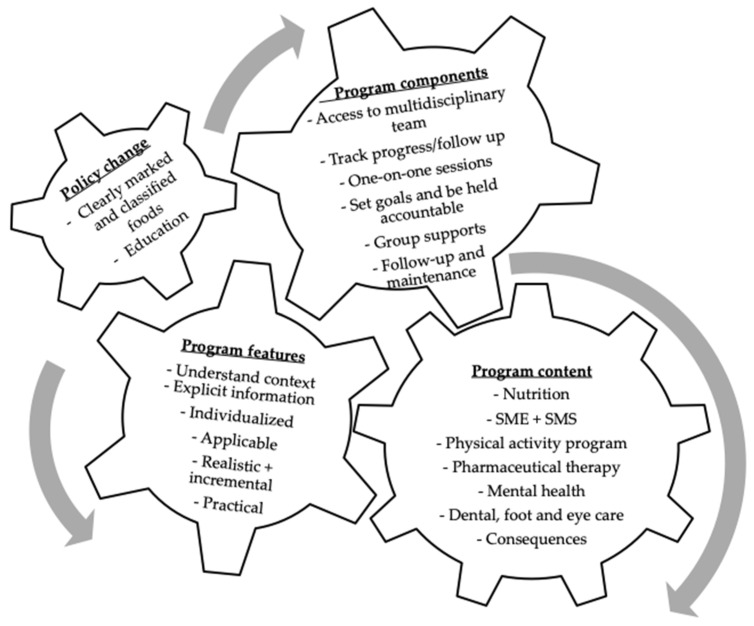
Program content and delivery specifications. Participants reported the information content as well as the program components and delivery mode.

This theme represents the main objective of our study. The discussion reflects what participants sought in a LI program, thus, helping to identify potential program content/subjects, features and components for future interventions (Figure 1).

##### Program Content

The programing requirements espoused by participants included training in a wide variety of components across all of the pillars of T2D management with specific delivery modes and program components to help enhance their success (Table 4). Learning the gravity and severity of consequences is something that they considered should be taught, “…in their courses and stuff, they should bring in videos and pictures of people who have had amputations or have had lost limbs because of their diabetes or illnesses or had surgery or almost died or—they should use that evidence to show the people who still have a chance what can happen to them if they don’t smarten up”—PT178. Additionally, how to pack an emergency kit, blood glucose monitoring, nutrition, eating on a budget and diet for all of the family within a household, foot care, and medication effects were requested content areas. Moreover, in regard to healthy eating and general diabetes care, participants explained knowing what they should not consume but required more knowledge of alternatives that they should/could consume; thus, questions related to what, how much, and when to eat should be addressed. This was also reflected when keeping track of their blood glucose, “previously my blood sugars had been when I initially started was taking my blood 3 times a day, right? With the prick, with the finger pricks, and, and I do that—I do that religiously and I would do it all the time. I never really understood what I was doing as I—as I said”—PT555.

##### Program Features

Delivery of information in programs should be “explicitly clear” (Table 4). The content provided should be applicable in daily life, practical, and realistic to address the participants’ needs, for example, some mentioned learning how to order something healthy from a fast-food chain, or what healthier options to look for at a drive-through window. Participants desired learning how to go from big portions to smaller portions or wean off sugar and processed foods; they felt that learning this would actually have an impact on their health, therefore, the information has to be very applicable to real life situations. In addition, learning how to use a glucometer and the importance and relationship it has with food consumption and exercise was an active learning approach they desired, reflecting the need to provide practically oriented information. Some participants explained that doing the measurements but not really understanding why or what they could do about it made it essentially useless (Table 4). Lastly, a continually expressed requirement from participants was to make eating healthy easy by means of, for example, providing a line of T2D-friendly fast food, having someone else provide the food and take care of the cooking, it has to be as, “simple as opening a bag of potato chips”—PT115; hence, realistic guidance should be presented.

##### Program Components

Programs with ongoing access to a multidisciplinary team, including registered nurses, RD, kinesiologists, pharmacists, and mental health therapy and counseling, were considered to be an asset. Participants requested a broad range of nutrition skills including cooking classes for T2D. Providing individualized exercise programs and understanding and knowing the specific needs of those enrolled were considered essential. Furthermore, participants described wanting to be held accountable if they didn’t comply with the program.

Individualized attention, follow-up contact, and ongoing group supports were also identified as critical components of a program. Participants explained when they had one-on-one sessions with HCPs who could answer questions, set goals, and track their progress led to plans that worked for them as well as a sense of purpose. Likewise, participants desired a program that was ongoing, providing regular refresher sessions, for example, annually or biannually, sending reminders via text message, e-mail, phone calls, or providing a coach as an incentive to keep going. Group support systems were a recurring request, with participants valuing being able to be part of a group of people that understood them, living through the same processes, and relating to their experience. Having a group that was ongoing, meets on a regular basis, and provides some type of buddy or mentor system was described (Table 4).

##### Policy Change

Lastly, some recommendations on a policy change level were made. A request for clearly marked healthy food spaces in grocery stores and clearly marked diabetes-friendly products in convenience stores was requested. Furthermore, learning more about health and nutrition at an early age would facilitate incorporation of healthy eating habits in adulthood. A perception was voiced that inclusion of healthy eating and food skills development within their school curricula would have improved their chances of maintaining healthy eating through their adult years (Table 4).

#### 3.1.5. Theme 5: Participant Advice

From participating in different programs, receiving advice and guidance from different sources, and through their own personal experience, participants are valuable sources of experience and knowledge, able to provide advice to help other people manage T2D. Thus, this final theme reflects the perspective of advice provided to people with diabetes from people with diabetes (Table 5). Tracking food consumption (limiting, measuring, monitoring, and logging), being mindful of food choices (do not add salt, no pop, do not eat what you do not love, eat seasonally, and consume vegetables), taking ownership of one’s health (have a plan, have a support system, and think of the long term), and having a good team support (health care team, friends, and family) were advice and recommendations suggested by participants to help other people with T2D.

## 4. Discussion

Our previous study identified a need to provide more guidance to enable people with T2D to be able to incorporate healthy eating behavior in their everyday lives [5]. The present study was designed to understand participants’ perceptions and lived experiences regarding healthy eating behavior change, and to identify and understand potential intervention components to guide the development of future LI programs.

Findings from the present study reflect participants’ perspectives that living with and managing T2D can be an overwhelming process associated with negative feelings, anxiety and, sometimes, low motivation to carry out self-care activities. Most of our sample (*n* = 10) was represented by an age group over 61years; thus, it is understandable that these participants’ motivations were partly to avoid losing their independence, which has been reported previously in older women [29]. Likewise, Crossley, through the use of focus groups, illustrated how health is connected to morality [30]; participants’ motivations to change was partly to avoid being a burden to others and to maintain their independence, corresponding with these results. Thus, it is understandable that age and independency are factors that promote motivation in older adults [31]. As noted by Atkins and Michie [32], our results reflect that healthy eating behaviors are influenced by and form part of a complex, evolving system. Participants indicated that their health, background, history, and their external environment influence their behavior in that work, family, and HCP promote changes in their behavior towards more positive or negative outcomes.

Participants had a good understanding of the relationship between T2D and eating habits. When participants were asked what healthy eating habits were, a wide variety of accurate information was described, referring to types of foods, nutrients, degree of processing, and perceived nutritional value. These findings are consistent with a previous study that explored healthy eating [33]. In line with previous results [34,35], a health behavior cycle was described consistently throughout the interviews, where participants reported going through cycles of “good behavior” and “bad behavior”. Qualitative studies have provided insight towards understanding this fluctuating behavior, for example, participants’ identities, social factors, resources, environment, and competing priorities influence eating behavior. A deeper description of these factors is beyond the scope of the present study, but further detail can be found in the review by Bisogni [36].

Even though the interview guide was developed to focus on healthy eating and how to facilitate adherence to the guidelines, programming requirements outside this scope were mentioned. The “ideal” program content, as informed by participants, should involve care that covers the major pillars of T2D care including SME + SMS; NT; PA; pharmaceutical recommendations; foot, eye, and dental care; as well as mental health, aligning with Diabetes Canada NT Guidelines [2] and reflecting on the complexity of T2D management [1]. In addition, several additional program components and specific program features were sought. In regard to the latter, active learning, hands-on approaches were desired for all of the knowledge being taught to aid in understanding and retention of information. For example, instead of just recommending or teaching what not to eat, participants would like to learn what they can eat, how to prepare it (cooking classes), and what it looks like in a real-life situation (e.g., drive through at a fast-food restaurant). Another example is when teaching the relationship between food, PA and blood glucose levels, the lesson should incorporate active real-life practice so that participants can visualize the outcomes of their actions. Fritschi (2019), through the use of content analysis, reported that a personalized supportive approach from the study team together with active learning and self-monitoring are factors that enhanced knowledge and improved health behaviors [37]. In addition, promoting personal discovery through self-monitoring (cause and effect) and/or patterns of associations (daily activities and changes in blood glucose levels) generates subsequent action planning resulting in sustainable behavioral change [38].

Interviewed participants emphasized the importance of researchers and HCPs learning who is in the program—understand context—because through this understanding program leaders, can provide individualized advice that will be effective for each participant. Research in this area has recently been developed by the 5As Team, which showed that patients with obesity want personalized, evidence-based care [39,40].

Thus, future programs for T2D management should consider incorporating the key processes for a personalized approach (convey compassion and listening, try to make sense of root causes and contextual factors, focus on whole-person health and action planning, and foster reflection and experimentation within others) to support and manage health improvements [41]. These concepts mirror the expressed needs of people with T2D in this study. This approach has been recently incorporated into the Canadian Obesity Guidelines, which conceptualizes a patient-centered health outcome approach [42]. Consideration should be given to adapting this approach for people with T2D. Along the same lines, future program developers should consider increasing the skills, abilities, and confidence of HCPs to individualize care, which could be achieved, in part, by training in healthy conversation skills, a technique that allows the development of competencies for identifying and creating opportunities to hold healthy conversations, using open-ended questions, reflecting on practice, listening more than talking, and supporting SMARTER (specific; measurable; action-orientated; realistic; timed; evaluated and reviewed) goal setting [43]. Such competencies would address needs revealed by themes induced from the participants’ interviews. Furthermore, an ongoing group or peer support system should be incorporated into programming. Such support groups may provide a safe space where empathy, acceptance, and understanding can be sought. Mohr et al. showed that these groups were more effective when moderators were integrated into the team [19] and, recently, such groups have moved to (or added) online interfaces though social media such as WhatsApp, Twitter, and Facebook [44]; thus, enabling interactions even when face-to-face meetings are not possible. This could be important for uptake, given the priority juggling challenges voiced by our participants.

Lastly, changes at the policy level were requested, including clearly marked “healthy options” spaces in convenience stores to facilitate healthy eating. Research in this area has advanced in recent years. Mexico implemented front-of-pack warning labels on food and beverage products deemed unhealthy to help people make healthier choices; however, it is too early to evaluate the effectiveness of this approach [45]. In Canada, research on how food environments shape the availability, affordability, and social acceptance of food and nutrition choices is growing. Nutrition report cards have been developed to assess the healthfulness of children’s food environments [46] and could be applied to key environments for people with T2D to support healthy eating in this population.

The present study comes with some limitations. A recent research study aimed at understanding how to facilitate healthful behavior change in a culturally sensitive self-management support program for T2D in the United Kingdom was undertaken [47]. Their approach consisted of applying the behaviour change wheel (BCW) in the design and identification of potential targets for intervention through conduct of focus groups and using a preconceived deductive analysis based on the BCW [47]. Key concepts of social support, credible information sources, and demonstration were identified as techniques helpful for behavior change. These themes were also voiced by our participants and suggest that information can be applied across situations where behavior change is desired. While the use of theory and framework is encouraged when designing health care interventions [33], and lack of a grounding framework could be seen as a limitation of the current study, our essentialist paradigm and inductive approach precluded us from using predefined codes, and thus allowed the perspectives of participants to be captured faithfully. Codes and categories were derived directly from the data, attaining methodological coherence. Another limitation of the present study refers to our sample. First, self-identified T2D inclusion criteria is a limitation since no proof was obtained. Furthermore, even though efforts were made to include participants from different cultural backgrounds, most participants were from a white ethnicity and medium-high socioeconomic background, hindering the generalizability of the results. Furthermore, the majority of our sample was in the age range over 51 years (*n* = 12), thus, our results represent the views of an older population that is, nevertheless, consistent with most people being middle- or older-aged at diagnosis. Lastly, although the sample size (*n* = 15) might be considered a limitation, there is no clear-cut definition of what constitutes an adequate sample in qualitative research. Sandelowski, suggested that a sample size allowing both deep analysis and diversity of experiences could define adequacy of numbers [48]; in the current study, many points of views, experiences, and suggestions were derived from the analysis.

## 5. Conclusions

In conclusion, T2D management is influenced by complex evolving factors in people’s lives. The voices and needs of people with T2D need to be incorporated into LI programs. The results of this study indicate that people living with T2D request more extensive, comprehensive, and ongoing support for changing health behaviors. Shifting of program content, delivery methods, and provision for long-term support is required to address participants’ needs and the multifaceted etiology of T2D. Taking these actions would start to address the knowledge–action gap and help people with T2D manage their disease over the long run.

## Figures and Tables

**Table 1:** Demographic characteristics of participants.

Variables		Mean (Min–Max)
Age (years)		61.4 (33–79)
Diabetes diagnosis (years)		12.6 (5–25)
		***n* (%)**
Gender	Female	8 (53)
	Male	7 (47)
Ethnicity	Aboriginal	3 (20)
	Black	1 (7)
	White	11 (73)
Education	High school or less	1 (7)
	More than high school	14 (93)
Employment status	Working	4 (27)
	Retirement	7 (47)
	Other	3 (20)
	Not reported	1 (7)
Household annual income	<$59,999	4 (27)
	>$60,000	9 (60)
	Not reported	2 (29)
Annual income	<Meet needs	1 (7)
	>Meet needs	13 (87)
	Not reported	1 (7)

Data presented as mean (min–max) or (%).

**Table 2:** Programs classified by their main pillar of T2D management.

SME + SMS	Nutrition Therapy	Physical Activity	Pharmacological Therapy
- Diabetes studies led by university researchers - Educational program for recently diagnosed diabetes at local hospitals or primary care clinics - Diabetes in pregnancy program - Conference	- Healthy eating program offered by a church - Counseling by a RD - Commercial weight loss programs	- Program led by primary care HCP - Activity “prescription” from MD - Personal trainer - Fitness program - Memberships at gyms or recreational centers	- Meeting with pharmacist - Medication adjustments by MD

**Table 3:** Representative quotes describing patient-oriented themes.

Theme	Example Quotes
**Theme 1:** Dealing with being diagnosed and living with diabetes	
Feelings	“Lots of anxiety. Sometimes I will just be like ‘why waste my time? I’m dying anyway.”—PT153
	“Doing a whole change like [DM management] … it’s overwhelming.”—PT300
Engagement	“I have a family history of diabetes. My father was diagnosed at approximately the same age that I was. Uh, he did not particularly look after himself that well, as a result of which he died at age 70 from diabetes complications. And I am determined that I am not gonna go the same way. I will do what I need to do to maintain my health as best as I can.”—PT364
	“People who have a reason to live take better care of their bodies than people who don’t have a reason to live. The fact that I have, uh, a grandson now that I adore and I’m more open to going back to the diet and exercise than I was 6 months ago or 6 years ago, that’s not coincidental. That’s just the way life goes.”—PT132
**Theme 2:** The impact that external forces have on T2D management	
Health care providers	“He was a specialist, first of all, and he was, like, following me really closely and we talked a little bit about things I could do, and he was giving me a purpose. ‘In six months, I want you to come back with lower numbers!’ I was like ‘okay. I’m terrified.’ But six months, I try whatever I can.”—PT153
	“My doctor just sent me to diabetes classes […], he had me meet with a nurse as well, every 3 months I believe, to go over, you know, like the—the health issues with the blood pressure, the feet. Stuff like that. So, uh… you know, he’s maintained this… you know, over the last 10 years. Like I go every year for this assistance with the nurse and he… you know, validates my blood work and stuff. So, like, I’ve had the help.”—PT149
	“Completely demeaning... it was painful and awful. And then you walk in to some guy’s office and he looks at—I have a log book of everyday, 5 times a day when I was stabbing myself with insulin, every unit—and he looks at me—he says ‘well, these aren’t the right numbers. I think you’ve got too many zeros on these.’ And I was like ‘I can recall every single stab. Every single time I had fluid pockets under my skin from injecting that much subcutaneous—like you can’t tell me that I’m lying or that I don’t know what I’m talking about.’ So it’s—it was pretty bad... And he sends me back with metformin which, you know, my other—my—my specialist had counseled me was not a good option for breastfeeding. So it was—it was a terrible time.”—PT623
	“So I never went back because she [RD] “This is what you can do. Solve your lunch problem and everything will be fine”. So I thought well how could I go back to tell her I don’t like the plan? It wasn’t working.”—PT38
Family	“I’m lucky that my wife still likes to cook and that she’s a really good cook and she likes flavorful food, which I do.”—PT70
	“My wife supports me. She supports 110% so I’ve got a very good support system.”—PT178
	“My wife still doesn’t understand what I should be eating. I—our diets are separate. I cook my own food. I… buy my own groceries. I eat separately. ‘Cause my wife—I don’t think really has an understanding of what I should or shouldn’t be eating and also it then becomes restrictive on what she can eat.”—PT555
	“I think that’s what a lot of people… that I’ve talked to are missing is that they’ve, you know, they’ve got the diabetes. They’re trying to make the changes. But you’re not eat—you’re not getting the support from your family. And if you’re the one that’s doing the cooking and providing the food and your family’s not willing to change to allow you to have a better diet then… it’s—it’s hard for you to do it.”—PT364
Life situation	“In my career I also travelled a lot and I think that was probably one of the onsets of diabetes was that I was travelling… in hotels, eating in restaurants continuously… eating very, very badly. Limited activity and bad food.”—PT555
	“I’ll buy groceries, I’ll buy this, I’ll buy the cauliflower, I’ll buy the—I like the stuff. And yet, I guess because what I use as an excuse there is I’m driving my husband places. I’m driving my granddaughter places. I’m going out to [grandson’s name]. I’m going out to look after [grandson’s name] a couple times a week. And I get home at 5 or 6 and I don’t feel like cooking much. So I mean it’s a lot easier to have, um… What do I usually… well order a pizza or something.”—PT38
	“The fact that back home I wasn’t really the one cooking...here, I had to cook for myself, which was a new experience [stammers] a—a really new experience for me, coupled with the fact that I had to use ingredients I wasn’t really familiar with.”—PT153
	“Our income is quite limited and a fair amount of stress exists in my head because of that and that affects what we buy, what we do and I’m trying to think of ways to economize and yet, uh, try to get better quality of vegetables, and yet, on the other hand, um, I think I’d like to have some more meat into our diet.”—PT115
	“Now with this Covid, and that’s a poor excuse, but it is a reality too, you know what I mean? Because I had tried to—I had joined the [name] Rec Centre on March 5th. I did one day. And then it was closed after that.”—PT149
	“A lot of people are feeling a strain of not being able to get out and about with all their friends and relatives and that, and that’s making everything more difficult for them.”—PT364
**Theme 3:** Understanding healthy eating behaviors for T2D management	
Nutrition knowledge	“It’s a balance of things, you gotta do your whole grains, you have to do your fruits, you have to do your vegetables. Um, naturally occurring… I guess sugars if you’re going to consume sugars like from fruit, from watermelons, the things that your body can digest, staying away from the manufactured to the man-made or the artificial stuff. So that’s where I think of… not—healthy eating. Rather than dealing with process, anything that’s natural.”—PT178
	“Healthy eating’ would be, um, an equal—you, know, making sure that you put your proteins in. You’re, um, making sure that most of your plate is vegetables and fruit as compared to carbs and proteins.”—PT159
	“Basically following Canada’s food guide. You know, that’s basically how you should do it.”—PT149
	“To me that would mean, um, sort of a mix of foods and—and kind of a balanced mix. So, uh, right amount of vegetables and fruits. Um, uh—um—the—the correct amounts or—or balanced amount of protein, carbohydrates, vegetables, fruits, um, combined with, you know, enough liquids to keep it all working.”—PT70
Behavior cycle	“There’s that initial transition on diagnosis and trying to—trying to get better control and better behaviors. And—and then there’s the—the kind of the long haul process of, um, trying to follow the guidelines and… the temptation is always around you, all the time. Um… And, you know, over time, I found that I able to… to—to stick with the rules but every once in while that just gets boring or, you know… I just can’t—I don’t want to do that anymore. I take a break for a day or a meal. Um, and then feel guilty about that and get back on track.”—PT70
	“I’ve just in to the habit of doing what I think I need to do and, you know, if I… feel that I’m not getting enough exercise, maybe I’ll just go out and do a little bit more or… you know… Yeah, occasionally I’ll eat probably what I shouldn’t eat but then [smacks lips] realizing I’ve done, I try to make the rest of the day or the next day that I’m eating more sensibly and I’m watching what I’m doing to kind of counter balance when I do sort of fall of the wagon.”—PT364
	“My problem, again, is night eating. Even getting up in the middle of the night to eat. So I could eat very healthy throughout the whole day and be within, um, the right category, um, of carbs, sugars, etcetera, and proteins, and then I would have my downfall in the middle of the night.”—PT159
	“Even though I know moving for health is—it’s good just to get out and do a walk… […] So sometimes you say ‘forget it. I’m just not gonna go.’ I become lazy and—and, you know, sedentary and decide not to do it, right?”—PT149

… Pause; […] ellipsis—quotes are slightly edited for readability. Participant (PT).

**Table 4:** Themes describing program content and characteristics as described by participants.

Theme	Example Quotes
**Theme 4**: Understanding needs and identifying intervention components	
**Program Content**	
Nutrition	“What’s in food? How do you read label? So, you know, getting to know that, um… you know, eating a—a bran muffin at Tim Hortons is the same as eating 4 slices of white bread with butter. You know, it was an eye opener.”—PT555
	“I’d like a diabetic diet that allowed a blowout once a week or something on stuff you really enjoy as opposed, it just feels like you’re following such a regimen and it’s just you never get to escape it. Or if you do you’re, you’re feeling guilty about it, but.”—PT115
Physical activity	“Some sort of exercise program where you can encourage people to maybe get together even if it’s just something as simple as walking. You know. Meet every few days, once a week or whatever and just… go for a walk somewhere where you can talk to each other. You’re doing the same activity, you can support each other and, you know, okay, well, you know, you’re getting tired. “Well, why don’t you go just a little bit farther and you’ll feel better type of thing.”—PT364
	“Exercise was not my fortitude. Still isn’t. So, like, maybe perhaps someone, uh, to go with me, to train me, that would work.”—PT153
Hands-on learning	“Help them understand […] something as simple as eating a meal, taking your blood sugars before, during, after, uh, then doing the same thing the next day but going for a walk for 15 min and seeing, you know, doing the same measurements and seeing the change. And it’s like ‘okay. Hmm. That—I—I can see it, you know. I can see what the combination of diet and exercise does for my blood sugars. Even on a short-term basis, so….”—PT70
	“Packing an emergency kit and what does that look like.”—PT132
Mental health	“Having a support system, uh, ack—acknowledging that there’s a, um, there’s a mental, uh, a mental side, um, helps fight the—the disease is really important, cause if you don’t address that… it’s not going to work.”—PT153
	“Having the mental health improved is… I think it’s primary. If you need to… If you’re gonna have success with something, I think you need to make sure your brain is working right.”—PT97
Foot care	“Foot care […] it’s important to always look after your feet, make sure that you don’t—when you put your shoes, that you shake your shoes out. You—you wipe the bottom of your foot off before you put your foot into the shoe. So she’s [nurse] very remindful of that because she knows that the extremities… you can, you know, if you’re stepping on a stone or something in your shoe that, you know, and you don’t feel it—as the diabetes progresses and you don’t feel it, you can get like a sore and sometimes that sore doesn’t heal.”—PT149
Consequences	“They mention, yeah, you’re gonna get heart disease, you’re gonna gain weight. This is gonna happen, yes you can lose a toe, a foot, that—but—but they don’t really show… pictures, they don’t make it graphic.”—PT178
**Program features**	
Understand context	“The idea of trying to… um… trying to understand who the participant and—and sort of where they’re coming from, what they’re… possibilities, opportunities, you know, lifestyle, um… their whole life situation is, uh… will make it easier to… to figure out… how they—what kind of changes they can make most readily that will, you know, be good changes for them to make.”—PT70
	“You gotta kind of understand what—you know, where people are at […] find people’s ‘whys.’ People have a ‘why.’ You just gotta find it and then relate it to them. And so that will help them… be on track for things.”—PT623
Explicit information	“An overall household plan for diet. But the biggest thing, and I’ll you that is really… very specific, you know, if you’re gonna down and have a breakfast, this is what a good breakfast it. This is what a bad breakfast is.”—PT555
	“So having someone help me, for example, going for the low-the glycemic index food. And explain how it works. That would be amazing. Because I go for the—I know what it is. Like I said I have a bachelor degree in science, but even that is not helping because I’m just overwhelmed with the fact that I’m the sick person […] Having someone going through the list again and explaining in light of… the dia—the diabetes I have, will be, actually, more effective. Why do you want have low glycemic index food in your—your plate? Uh, how much of this do you want to have? That would be great.”—PT153
Individualized	“Getting, um, uh, oh, like a guideline, do you know what I mean? As to what I should be eating, how much I should be eating or what—what—how much amount of carbohydrate, protein, and fat and—and—that I should be having at my age and my weight.”—PT149
	“The North American diet’s changed so much and as Indigenous people, that was not our diet. Uh, I mean, before contact, you know, we had a very specific diet and probably a very limited diet. And with the in-introduction to refined foods and such, it’s… it’s been really, really bad for Indigenous people so… what is that dietary pattern? What it is? Should—when should we be eating? And what should we be eating.”—PT364
Applicable	“Kind of train people to look for what that looks like at the drive through window at Tim Hortons or McDonalds.”—PT132
	“Teaching them how to go from very big portions to smaller […] learning to wean yourself off of your portion sizes.”—PT178
Realistic and incremental	“I think what—what maybe help—would help people is instead of just doing a… a whole—well, we gotta change from here to here, um, we need to change your—basically your whole eating habits, maybe—maybe just start ‘okay, we’re gonna try… no pop for these two weeks’ and then ‘okay, you can do that. Alright. Um… the next 2 weeks, let’s not have hamburger. Or if you gotta have hamburger, make it lean’ or… you know, that kind of thing. Uh, ‘eat chicken instead of beef.’ Um… do incremental steps maybe, um… instead of doing the whole—the whole… the whole big change.”—PT300
	“What’s feasible for you to do without demanding some extraordinary effort or some kind of effort that’s—that’s really not natural to you. That—that is too significant a change for you to make. Maybe by taking a smaller step, you may eventually achieve a larger set of steps, but, um… So I—to me, I think that’s—that’s part of the core of the practicality of some programs, is that, you know, while holding out sort the ideal… um, showing people that you can—you can at least make progress towards the ideal by taking a small step or two small steps, or you know… Along that line.”—PT70
Practical	“Getting the cognitive understanding or practicality in regards to that instead of just saying well you should just have less. Well then, with the program of feeling full they said have a hamburger, but cut it in half and have half now and have half later. That was a practical application of exactly what you should do and how to do it. Right?”—PT87
	“Maybe you should come up with a line of diabetic fast foods. There’s my assignment for you.”—PT115
**Program components**	
Access to multidisciplinary team	“Having access to the nurse, from time to time, with this program, it was really good, because now I—I had someone I could actually ask questions from time to time. So that did make the experience a little bit easier to go through.”—PT153
	“When I talked about the pharmacy… uh, oh, at [name of pharmacy], that was one of the best sessions because it was a very—it was one to one. It was open. And as the pharmacist said ‘ask any question. There’s no dumb questions here.’ So I really began to ask, you know… a lot of questions and he had a much more, uh, layman explanation for things—simple things.”—PT555
Track progress/ follow up	“I really do need a coach in a way. I don’t know how assertive the coach has to be with me but just something to remind me or to give me the incentive to keep going.”—PT115
	“Having a support to call and say ‘ok, I’m struggling with this.’ ‘How do I… you know, I can’t figure out how to do this with this particular item.’ You know? And I also got this and this on it. You know?”—PT159
One-on-one sessions	“Somehow we still deal with what we should be doing and maybe more one-on-one time, going over it and asking where we were struggling. What is it, you know, um, allowed us to not complete it? Or just the follow through on it all. I mean, do we have to be babysat? Again, I’m thinking this is an older group that you’re working with, more so, and, um—I don’t know, maybe we need more hand-holding.”—PT159
	“How have you changed because of the information you’ve gotten? What have you been able to stick to? What do you have trouble with? And let’s, you know, let’s take another step.’ You know? Or I would even, um, [smacks lips] you know, at first, maybe just—let’s just talk breakfast for a week, right, like just revamp breakfast and then ‘what did you like? What sticks with you?’ You know, it’s just—it’s just that learning isn’t a one-time thing. It has to evolve and change. You know?”—PT555
Set goals and be held accountable	“Maybe having them set goals? Beforehand and be accountable. And then check in with them, like, ‘why? This is what you wanted. Why—you know, when you’re not here, what do we need tweak, what do we need to change? Do you need to do a repeat? Do you need to, you know…’ but have something, cause in the beginning they will be motivated.”—PT159
	“Not getting off so easy if you were supposed to go home and do homework and you came back without—‘oh, I didn’t have time’, you know […] Penalize us. Charge us. Charge us for the course. It’s free but unless you don’t do it, then you have to pay back.”—PT159
Group support	“I think people are incredibly lonely. I think there is a case to be made for, um, a support group that doesn’t stop. A support group that genuinely cares about one another.”—PT132
	“Maybe even sort of a—a mentorship or a one-on-one where somebody that’s had diabetes for a few years can kind of look after or talk to somebody that’s just starting on the—the—the journey through and can let them know that, you know, some of the things that they’re gonna find, some of the problems they may have and that just… I guess let them that there’s somebody there that can help them or just—just talk to them and support them.”—PT364
Follow-up and maintenance	“I wish they had continued on with them [diabetes classes] and doing them because I think taking something like that every couple of years as sort of a refresher just helps kind of confirm that you’re doing what’s right and, you know, the—you know, the recommendations are changing over time so to make sure you’re doing what they’re recommending nowadays, you do and opposed to what they recommended 5 years ago.”—PT364
	“Follow-up, partnerships, groups. It’s the same as dealing with a, a 12-step program. You keep on going to meetings to reinforce. And that’s what [name of commercial program] uses. Right? The support systems.”—PT87
**Policy change**	
Clearly marked food items	“To have, uh, from the side of the people that are running these stores, spaces that are clearly marked with the good choices, with the good snacks, uh to do something on that side, so that people know this is a good choice. You know, even if it’s a, you know, seal of approval or something from somebody that says this a good thing to eat [...] Have an awards program for the, the, uh, the convenience stores of who does it and how well they do it, and to actually give them some kind of trophy or cash-incentive or something for their good work towards, you know, the health.”—PT132
Education	“If you can get young people where they can get that education, it—it probably is going help them a lot more than… […] just, you know, us talking about it all the time. If you’re learning it when you’re young… […] then you got a better chance of following through later in life.”—PT364

… Pause; […] ellipsis—quotes are slightly edited for readability. Participant (PT).

**Table 5:** Themes providing advice by participants to participants.

Theme	Example Quotes
**Theme 5**: Participant advice	
Healthy habits	“Try and reorganize or recognize your, uh, your stomach as your basic guide and things like eating what you want but only eating half the amount. Things like that where you’re recognizing when you’re full, and uh, that most of the time you’re eating when? you’re not hungry.”—PT87
	“We will sit down and come up with ideas about a weekly menu and sort of what we’d like to eat.”—PT70
	“Anything that you can maintain for the rest of life is the kind of change that you want to make.”—PT623
	“I, uh, have gone away from that, uh… sort of, uh, empty calories and such, like I said, I don’t eat in the evenings anymore. So don’t sit in front of the TV and eat potato chips or anything like that.”—PT555
	“Do the medical things, have your eye exam and your… you know… Uh, exam for your… your kidney—your kidney levels and all that sort of stuff and, you know, if they recommend doing it once a year, then try and do it once a year so that if you are gonna start developing problems, you’re—you’re getting ahead of the game and maybe you can prevent them from getting really bad.”—PT364
	“I honestly think… I’m overweight, for the most part of—because of the… size of portions I was having. Um… so I… watched the… quantity I’m actually eating. And… I… I had a bad habit of having soda for super. Um… so I don’t have that anymore and I have—try having water. Um… and yeah. Um… I try to… instead of just having meat and—and pasta and that kind of stuff I try to have a side of salad or vegetables or something like that to go along with the meal […] trying to have fresh vegetables around.”—PT300
	“My wife, when she quit smoking, she started doing the dishes after supper because normally she would finish the meal, go sit on the front porch or whatever to have a cigarette and then clean up. She changed that habit from instead of smoking after supper, she does the dishes, right away. She gave up smoking because she replaced the smoking with doing dishes. Do something to replace the time when you eat. Find out when you always wanna snack, and when you feel that urge, do something. Uh, I don’t know, take up a hobby, do puzzles, do… uh, crosswords, whatever.”—PT178
Healthy eating	“Eliminate my snacking after supper and changing my meal pattern from largest at supper to the largest meal at breakfast. Or lunch.”—PT178
	“Less pop. You know, well, no pop actually, I shouldn’t say less pop. No pop. Carbonated water instead.”—PT159
	“Try to eat seasonally.”—PT70
	“Use the whole grains. Even if I go out, I ask for a whole grain bun.”—PT149
	“Lots of water. Lots of water.”—PT132
	“Just try and remember, eat veggies.”—PT115

… Pause; […] ellipsis—quotes are slightly edited for readability. Participant (PT).

## Data Availability

Data are available upon reasonable request to the author for correspondence.

## Supplementary Materials

The following are available online at https://www.mdpi.com/article/10.3390/nu13072301/s1, Supplementary File S1: COREQ: 32-item checklist, Supplementary File S2: Telephone recruitment script, Supplementary File S3: PPLP—One on one Interview guide, Supplementary File S4: Demographic questionnaire.
