# Supplementary material for: Contextually Appropriate Tools and Solutions to Facilitate Healthy Eating Identified by People with Type 2 Diabetes
Source: Nutrients. 2021 Jul 3;13(7):2301. doi: 10.3390/nu13072301 (PMC8308352; doi:10.3390/nu13072301)
Supplement: Supplementary file 1 [file nutrients-13-02301-s001.zip › Final V_Jun 2, 2021_Supplementary File 1.pdf]

Supplementary File 1. COREQ: 32-item checklist

Manuscript: 'Contextually appropriate tools and solutions to facilitate healthy eating identified by people with type 2 diabetes"

Consolidated criteria for reporting qualitative studies (COREQ): 32-item checklist

Developed from: Tong A, Sainsbury P, Craig J. Consolidated criteria for reporting qualitative research (COREQ): a 32-item checklist for interviews and focus groups. *International Journal for Quality in Health Care*. 2007. Volume 19, Number 6: pp. 349 – 357

Supplemental Table S1. COREQ Checklist

**No. Item**	**Guide questions/description**	**Location in manuscript**
**Domain 1: Research team and reflexivity**		
Personal Characteristics		
1. Interviewer/facilitator	MCAH conducted all interviews.	Methods: line 121 - 122
2. Credentials	RD, M.Sc, PhD	Methods: line 121
3. Occupation	At the time of the interviews MCAH was a PhD candidate at the University of Alberta.	Methods: line 121
4. Gender	Female.	Methods: line 121
5. Experience and training	At the time of the interviews MCAH had a background in Nutrition and Dietetics and a Master's degree in Medical Sciences. MCAH had previously conducted and participated in focus groups with participants with type 2 diabetes from Sherwood Park Primary Care Network as part of a previous research study from the University of Alberta.	Methods, line 121 - 122
Relationship with participants		
6. Relationship established	No.	-

7. Participant knowledge of the interviewer	Participants were briefed on the purpose of the study and understood the study was part of a research study for MCAH PhD.	Methods: line 123 - 124
8. Interviewer characteristics	MCAH was a PhD candidate during the time of the interviews and a junior researcher.	-
Domain 2: study design		
Theoretical framework		
9. Methodological orientation and Theory	Thematic Analysis according to Braun and Clarke (2006).	Method, line 138 - 143
Participant selection		
10. Sampling	Purposeful sampling was used. This method allowed for the selection of information-rich cases from which we could learn, yielding insights and in-depth understanding. Additionally, participants were recruited from different age groups, gender identifications, socio-economic backgrounds, and cultural affiliations to maximize variation of participant context	Method, line 56 - 66
11. Method of approach	Participants were recruited from the 5AsT cohort and the Alberta Diabetes Institute Research Contact Registry. In both cases, the original databases were searched to identify possible participants. Once identified, those from the ADI Research Contact Registry were sent information about the study by an administrator and asked if they were interested in participating. The list of possible participants was then provided to MCAH who approached them via telephone, as were individuals from the 5AsT cohort. In this first contact, MCAH explained the purpose of the study in more detail, as well as the procedure and time requirements, then answered questions and invited participants to participate (**Appendix 2**). Those interested were provided with an information letter containing all the details of the study and researchers' contact information.	Method, line 79 - 88

12. Sample size	15	Method, line 92
13. Non-participation	A total of 82 potential participants were obtained from the two databases, 68 were phoned, 29 did not answer and a voice message was left, 39 were invited to participate, 10 declined due to lack of time, 14 agreed but later declined to participate due to COVID-19 concerns or difficulties connecting to the video-conference interview; thus 15 consented to participate	Method, line 88 - 93
Setting		
14. Setting of data collection	The study took place in the Human Nutrition Research Unit at the University of Alberta, Edmonton, AB, Canada (prior to April 2020) and online using secure, encrypted videoconferencing software (necessitated by COVID-19 restrictions).	Method, line 70 - 72
15. Presence of non-participants	Two nutrition undergrad student were present for two interviews (only one per interview).	-
16. Description of sample	A total of 15 participants aged 30 – 79 years were interviewed (mean age 61 years). Participants had on average 12.6 years of T2D diagnosis varying from 5 to 25 years. Participants also varied in gender, ethnicity, education and socioeconomic status.<br>(see Table 1: General Characteristics of the Study Participants)	Results, line 162 - 166
Data collection		
17. Interview guide	Interviews were semi-structures using an interview guide (Appendix 3).	Method, line 128<br>Appendix 3
18. Repeat interviews	No	-
19. Audio/visual recording	Interviews were audio recorded (with participants' consent).	Method, line 124 - 125
20. Field notes	Journal notes were taken during and after each interview to record the researcher's reflections, feelings and interpretations.	Method, line 118 - 120

21. Duration	Interview durations ranged from 30 – 75 minutes	-
22. Data saturation	In this study, data saturation was not obtained since maximum variation purposeful sampling was used to capture a lot of different experiences.	-
23. Transcripts returned	No	-
**Domain 3: analysis and findings**		
Data analysis		
24. Number of data coders	To ensure rigor, two independent coders (MCAH, CBC) coded four interviews using open coding, enabling the creation of initial codes from which two independent coding manuals were created. MCAH and CBC compared and discussed the similarities to create a final coding manual.	Method, line 143 - 146
25. Description of the coding tree	The rest of the interviews were coded by MCAH, providing full and equal attention to each transcript and refining codes as needed. MCAH defined categories and subsequently checked for convergence and divergence with CBC to create the final categories.	Methods, line 146 - 149
26. Derivation of themes	Inductive approach was used, thus, no predefined outcomes were established, and instead, the coded categories were delivered directly from the data.	Methods, line 140 - 142
27. Software	CONSENTIA Inc. was used as a transcription service to transcribe verbatim audio-recorded interviews. They were then uploaded onto NVivo 10 (QSR), which was used to facilitate coding.	Method, line 131 - 137
28. Participant checking	No	-
Reporting		
29. Quotations presented	Yes, specific quotations were presented to help illustrate themes and provide examples. Quotations were identified by a participant number	Result, line 168 - 404
30. Data and findings consistent	Yes	Result, line 168 - 404

31. Clarity of major themes	Table 2, table 4, table 5.	Result, line 168 - 404
32. Clarity of minor themes	Table 2, table 4, table 5.	Result, line 168 - 404
