# Supplementary material for: Contextually Appropriate Tools and Solutions to Facilitate Healthy Eating Identified by People with Type 2 Diabetes
Source: Nutrients. 2021 Jul 3;13(7):2301. doi: 10.3390/nu13072301 (PMC8308352; doi:10.3390/nu13072301)
Supplement: Supplementary file 1 [file nutrients-13-02301-s001.zip › Final V_Jun 2, 2021_Supplementary File 3.pdf]

Supplementary File 3. Interview guide

PPLP - One on one Interview guide

1. **CONSENT**
   - In advance
2. **INTRODUCTION**
   - Introduce yourself and the study clearly
   - Background and Study objective.
3. **CONFIDENTIALITY**

   Remind the participants
   - Participation is entirely voluntary.
   - We will be audio-recording the interview.
   - You are free to stop the recorder at any time.
   - You are free to not answer any questions you don't feel comfortable with.
   - Personal information will not be disclosed, and it will be removed from the report. You will remain anonymous.
   - You are free to withdraw your consent and if you wish to have your data removed from the study, you may do so up until data analysis has started. That is 3 days after your interview.
4. **GROUND RULES**
   - I want you to do the talking; share as much information as you want.
   - There are no right or wrong answers; I want to hear a wide range of opinions.
   - What is said in this room stays here; I want you to feel comfortable sharing sensitive issues.
5. **INTERVIEW**

- See question guide

6. **CLOSURE**

- So this brings out interview to an end. I'd like to thank you very much for taking part in this research project. We appreciate your time and the valuable information you have shared with us today.

7. **Travel expenses**
8. **Gift**

- Pure Prairie Eating Plan

INTERVIEW GUIDE

START INTERVIEW

- Ice-breaker: please could you briefly introduce yourself and tell me a bit about yourself. How long have you been diagnosed diabetes?
- Record participant´s NAME, AGE and YEARS WITH T2D.
-

PART 1- Diabetes-friendly eating habits

We want to understand the lived experience of adults living with Type 2 Diabetes (T2D), specially their experience with eating habits.

1. **What does 'healthy eating habits' mean to you?**
2. **How important is healthy eating to you when you think about your diabetes?**

PART 2 – Understanding past experiences*:*

I want you to think about, and

3. **Describe to me, any strategies you have use to help you eat healthy in the past**

Prompt - What specific changes have you used or have made to help you eat healthy?

From these experiences that you mentioned,

4. **Can you describe to me why you think they worked or did not work?**

Prompt - What specifically helped you?

Was there something that trigger you at this time or motivated you to adopt this change?

What have you found difficult about adopting these strategies?

Out of all of the programs or people that helped you,

5. **Who or what has helped you the most and in why?**

Prompt - What did they do that was most helpful?

What strategies or tools were helpful?

PART 3 – Key messages

For the last part of this interview, I am going to present to you a summary of the Key messages from the Diabetes Canada Nutrition Guidelines.

1. *Select whole and less refined foods instead of processed foods, such as sugar-sweetened beverages, fast foods and refined grains*
2. *Reduce caloric intake to achieve and maintain a healthier body weight*
3. *Pay attention to both carbohydrate quality and quantity*
4. *Select unsaturated oils and nuts as the preferred dietary fats*
5. *Choose lean animal proteins. Select more vegetable protein*

We are working on designing a program that would help people with T2D incorporate these guidelines to their everyday life. I want you to think about

6. What would you need to be able to follow these guidelines?

Prompt - What would help you be successful at following these guidelines?

What in your life, or what around you would need to change to allow you to make that change?

7. What type of activities could we offer to help people make these changes?

Prompt – What would help you to follow these guidelines?

Prompt - What would you need to allow you to make these changes?

8. What are the difficulties you foresee in being able to follow these recommendations?

Prompt - How is it going to get difficult?

9. What would you need to be able to overcome some of these barriers?

Prompt - From the health care system, in your personal life?

10. What would you need to follow these guidelines for the long term?

Prompt - What if anything would you like to try in the future?

PART 4 – SUMMARIZE

Summarize, ask the participant if the summary is correct or needs to be changed, or if they would like to add anything else. Thank them for their participation.
