# Supplementary material for: Contextually Appropriate Tools and Solutions to Facilitate Healthy Eating Identified by People with Type 2 Diabetes
Source: Nutrients. 2021 Jul 3;13(7):2301. doi: 10.3390/nu13072301 (PMC8308352; doi:10.3390/nu13072301)
Supplement: Supplementary file 1 [file nutrients-13-02301-s001.zip › Final V_Jun 2, 2021_Supplementary File 2.pdf]

Supplementary File 2. Telephone recruitment script

Project Title: **Contextually appropriate tools and solutions to facilitate healthy eating identified by people with type 2 diabetes**

Outgoing Phone Call:

1. Hello, may I speak with ________________?

If recruited from 5Ast:

2. My name is [name of caller] and I'm calling from the 5AsT Study that you are a part of. It's the study about the evaluation of your weight management, that took place at the [Name of] Primary Care Network.

3. As part of a partner study, we are interested in learning more about patients' views on healthy eating.

4. At your initial consent in the 5AsT study, you agreed that we could contact you about future studies.

5. The reason I am calling is that we would like to work with you to learn about your views on diabetes-friendly eating habits, what healthy eating looks like in your everyday life and what would help people adopt diabetes-friendly eating habits.

If recruited from Alberta Diabetes Institute:

1. My name is [name of caller] and I'm calling from the University of Alberta as part of the Alberta Diabetes Institute.

2. You answer a survey and show interest in our study about patients' views on healthy eating.

3. The reason I am calling is that we would like to interview you to learn about your views on eating habits, why healthy eating is difficult or easy, and what would help people adopt healthier eating habits.

4. So, if you are interested in participating in this research interview, I can tell you details about the research process.

[to the best of knowledge and ability address patient's concerns and/or queries]

➢ *If patient agrees to hear more about the sub-study and the interview process, then continue with the following section.*

➢ *If patient does not wish to hear more, thank them for their time and attention.*

Background and Purpose of Sub-study

As mentioned, we are interested in learning about your views on diabetes-friendly eating habits, what healthy eating looks like in your everyday life and what would help people adopt diabetes-friendly eating habits.

The study includes one-on-one interviews and a focus group. The procedure would look like this:

- First, you would meet a researcher form the [University name] for a one-on-one interview for about 45-60 minutes.
- All the interviews will be audio recorded and typed up.

Both audio recordings will be uploaded and stored on password-protected computers. Only research team members will have access to them.

Please know that your responses will remain confidential and will not be linked to any individual identification.

As an appreciation for giving us your time, we are **offering a copy of the Pure Prairie Eating Plan book which you can use as a guide for a healthy diet.** We will also cover any parking expenses that you may incur through your participation in this sub-study. You will receive this honorarium at the end of your first one-on-one interview.

As a reminder, your participation in this sub-study is voluntary. You have the right to end the interview at any point and, decline to answer any question.

Are you interested in participating in this research?

[If not, address any questions. Then thank patient for their time and attention]

[If yes, then continue with the following information.]

Scheduling initial interview

The interview will take place at the Human Nutrition Research Unit at the University of Alberta. The address is 116 St & 85 Ave, Edmonton, AB. The unit is on the second floor room 2-004 at Li Ka Shing Center.

Dates and time available for the interview session are:

The person interviewing you is named [name of interviewer].

If you have to reschedule your appointment, please call **[phone number]** [please have the participant write this information down].

We will provide you with a reminder phone call about your appointment. However, if you'd like, I can also email you the information we covered.

Do you have any further questions?

Thank you for your time and we look forward to seeing you *(confirm time and date).*
