# Supplementary material for: Contextually Appropriate Tools and Solutions to Facilitate Healthy Eating Identified by People with Type 2 Diabetes
Source: Nutrients. 2021 Jul 3;13(7):2301. doi: 10.3390/nu13072301 (PMC8308352; doi:10.3390/nu13072301)
Supplement: Supplementary file 1 [file nutrients-13-02301-s001.zip › Final V_Jun 2, 2021_Supplementary File 4.pdf]

Supplementary File 4. Demographic questionnaire

Please write or mark the appropriate answer for the following questions.

- Date (mm/dd/yyyy): ________________
- Age: ___________________
- Date of birth: _______________
- Gender: Male / Female
- Years with diabetes diagnosis:__________________
- **Ethnicity:**

Please circle the appropriate answer(s).

- White
- Chinese
- West Asian (e.g. Afghan, Iranian)
- Japanese
- Korean
- Aboriginal (e.g. First Nations, Metis or Inuit)
- Black
- Arab
- South Asian (e.g. East Indian, Pakistani, Sri Lankan)
- Filipino
- Latin American
- Southeast Asian (e.g. Cambodian, Indonesian, Vietnamese)
- Other (   )

1. **For the following questions please put a checkmark in the appropriate answer**

   Education:

- Less than high school
- High school graduate
- Some college or university (have some post secondary education, but notcompleted)
- College or university graduate or above

Employment:

- Wages and salaries
- Income from self-employment
- Retirement income (pensions, old age security and GIS, etc.)
- Unemployed (not including retirement)
- Other ( )

Household annual income:

☐ < $ 10,000 if 1 to 4 people
☐ < $ 15,000 if ≥ 5 people

☐ $ 10,000 to $ 14,999 if 1 or 2 people
☐ $ 10,000 to $ 19,999 if 3 or 4 people
☐ $ 15,000 to $ 29,999 if ≥ 5 people

☐ $ 15,000 to $ 29,999 if 1 or 2 people
☐ $ 20,000 to $ 39,999 if 3 or 4 people
☐ $ 30,000 to $ 59,999 if ≥ 5 people

☐ $ 30,000 to $ 59,999 if 1 or 2 people
☐ $ 40,000 to $ 79,999 if 3 or 4 people
☐ $ 60,000 to $ 79,999 if ≥ 5 people

☐ ≥ $ 60,000 to $ 79,999 if 1 or 2 people
☐ ≥ $ 80,000 to $99,999 if ≥ 3 people

☐ ≥ $ 80,000 to $ 99,999 if 1 or 2 people
☐ ≥ $ 100,000 if 1 or 2 people
☐ ≥ $ 100,000 if ≥ 3 people

2. **Financial situation**: (How would you describe your financial situation?)

- I can meet my needs and still have enough money left to do most of the things I want
- I have enough money to meet my needs and to do many of the things I want if I budget carefully
- I have enough money to meet my needs but have little left for extras
- I can barely meet my needs and have nothing left for extras
- I am solely responsible for my treatment financial
